# Among the swedish generation of adolescents who experience an increased trend of psychosomatic symptoms. Do they develop depression and/or anxiety disorders as they grow older?

**DOI:** 10.1186/s12888-022-04432-x

**Published:** 2022-12-12

**Authors:** F. Giannotta, K. W. Nilsson, C. Åslund, P. Larm

**Affiliations:** 1 Division of Public Health, School of Health, Care and Social Welfare, Malardalen University, Box 883, Västerås, Sweden; 2 Department of Public Health, Stockholm University, Albanovägen 12, 114 19, SE-106 91 Stockholm, Sweden; 3 Centre for Clinical Research, Uppsala University, Västmanland County Hospital Västerås, S-72189 Västerås, Sweden; 4 Department of Neuroscience, Uppsala University, Uppsala, Sweden; 5 Department of Public Health and Caring Sciences, Uppsala University, Uppsala, Sweden

**Keywords:** Psychosomatic symptoms, Somatic symptoms, Musculoskeletal symptoms, Depression, Adolescents, Anxiety

## Abstract

**Background:**

Despite an increase in mental health problems, with psychosomatic symptoms having been observed in new generations of Swedish youth, the extent to which these problems correspond to an increase in adult mental problems is unknown. The present study investigates whether Swedish adolescents with high levels of psychosomatic symptoms are at risk of developing depression and anxiety problems in adulthood and whether sex moderates any association. Moreover, we aim to understand whether different clusters of youth psychosomatic symptoms – somatic, psychological and musculoskeletal – have different impacts on adult mental health.

**Methods:**

One thousand five hundred forty-five Swedish adolescents – aged 13 (49%) and 15 (51%) – completed surveys at baseline (T1) and 3 years later (T2); of them, 1174 (61% females) also participated after 6 years (T3). Multivariate logistic models were run.

**Results:**

Youth with high levels of psychosomatic symptoms had higher odds of high levels of depressive symptoms at T2 and T3. Moreover, psychosomatic symptoms at T1 predicted a high level of anxiety symptoms and diagnoses of anxiety disorders at T3. When analyzed separately, musculoskeletal symptoms predicted higher odds of having high levels of depressive symptoms at T2 and T3 while somatic symptoms predicted high levels of anxiety symptoms at T2. Moreover, somatic symptoms at T1 predicted diagnoses of depression and anxiety disorders at T3. Sex did not moderate any of the relationships.

**Conclusions:**

The study supports the idea that an increase in mental health problems, such as psychosomatic symptoms, can seriously impact the psychological health of new generations of young adults.

## Background

In the last few decades, there has been an observed increase in adolescents´ mental health problems. In a systematic review, Bor et al. [1] pointed out that while externalising problems were rather stable during the transition between the 20th and 21st centuries, internalising symptoms increased, especially among adolescent girls. Consistently, cross-cohort comparison studies, conducted in the UK, Greece, Iceland, the Netherlands and Norway, showed a long-term increase in adolescent emotional problems from 1970 to 2000 [2–5]. These trends were only partly confirmed in the period between 2000 and 2018, when the increase in mental health problems, especially psychosomatic symptoms, concerned mainly the Nordic countries [6–8]. All in all, these studies support the idea that the mental health of new generations of youth, specifically those living in the Nordic countries, may become a more serious public health concern than it has been in the past.

Despite some increase in adolescent mental health problems, especially in the Nordic countries, the extent to which the observed youth mental health problems evolve into subsequently diagnosed disorders, such as depression or anxiety, in adulthood is still unknown. In this regard, Baxter et al. [9] have demonstrated that depression and anxiety disorders in adults did not increase during the period between 2000 and 2010. The authors have concluded that the observed increase of some mental health problems in youth might be explained by an increase in awareness of mental health rather than a real increase in mental health problems in adolescence. However, the discrepancy between the rates of youth mental health problems and adult depression and anxiety disorders might also be due to the types of measures used to assess youth mental health problems. Most studies, especially in the last two decades, have not used standardized measures of depression and anxiety, but have focused on psychological distress and psychosomatic symptoms/complaints [5, 6, 8, 10–12]. Consequently, the fact that mental health disorders did not seem to increase in adulthood can be explained by a weak causal/prospective link between youth psychosomatic symptoms and late adolescent/young adult depression and anxiety disorders. Therefore, in order to investigate whether the observed increased trends in some mental health problems, e.g. psychosomatic symptoms, that occurred among adolescents in the last two decades, lead to mental disorders, such as depression and anxiety, as they grow older, studies that focus on similar psychosomatic symptoms and follow adolescents into adulthood are needed.

To what extent are psychosomatic symptoms in adolescence related to the development of depressive and anxiety disorders in adulthood? A clear relation has been extensively demonstrated in cross-sectional studies (for a review, see [13]). However, given the interrelation between mental health problems – i.e., psychosomatic symptoms, depression and anxiety – cross-sectional studies are limited in that they cannot establish what predicts what. In other words, the extent to which adolescent psychosomatic symptoms predict the development of depressive and anxiety disorders in adulthood cannot be understood with a cross-sectional design. Consequently, to disentangle the relation between psychosomatic symptoms and mental health problems, longitudinal studies are needed.

There are some reasons to hypothesize a longitudinal/causal relation between psychosomatic symptoms and depression and anxiety. For instance, it has been suggested [14] that psychosomatic symptoms might affect the cytokine system, and that an inflammatory-based pathways might explain the link between somatic symptoms and the development of emotional disorders [15]. Another explanation is based on the effect of pain on the children social life, as it has been summarized by Beck et al. [16]. Psychosomatic symptoms are often associated with problems with peers, school absences, and academic failure, which are all linked with increased risk for depression and anxiety in adulthood [17, 18]. Nevertheless, to our knowledge, longitudinal studies with population samples that examine the extent to which youth psychosomatic symptoms are related to adults´ psychiatric disorders are scarce. Moreover, some of these studies, which do not control for baseline levels of psychiatric disorders (e.g., [19]), cannot give conclusive answers in any case. Among the few exceptions, Bohman et al. [14] and Shanahan et al. [20] have reported that somatic symptoms, such as headaches, stomachaches, or muscular/joint aches in adolescence or childhood, predict psychiatric/mental disorders in adulthood, while Shelby et al. [21] have found that functional abdominal pain in adolescence is related to anxiety in early adulthood. However, these studies focus on specific somatic complaints, while most of the studies that show an increase in youth mental health problems include a more comprehensive set of symptoms, both psychological and physical, in line with the definition provided by WHO [22]. Therefore, in order to investigate whether the observed increase in some mental health problems, e.g. psychosomatic symptoms, among adolescents in the last two decades might lead to increases in late-adolescents-young adults´ mental disorders, studies that focus on similar psychosomatic symptoms and follow adolescents into adulthood are needed.

In this study, we focus on a cohort within the recent generation of Swedish adolescents that have shown an increase in psychosomatic symptoms and follow them into young adulthood. Given the concern raised about increased psychosomatic symptoms, our primary goal is to investigate whether elevated levels of psychosomatic symptoms in adolescence are related to subsequent depressive and/or anxiety disorders in young adulthood. Moreover, as previous research has highlighted the finding that somatic problems increase more in girls (e.g., [6, 8]), we investigate whether psychosomatic symptoms elevate adult mental health problems in different ways in girls and boys. Finally, given that some of the above-mentioned studies have used only self-report measures to assess psychiatric disorders [20] while others have used diagnostic instruments [23], and that comparison between them is difficult, we used both self-report validated instruments and official diagnoses of disorders to increase the construct validity of the study. In short, using two cohorts of adolescents, 13 and 15 years-old at baseline, the aims of the study were:

1. To investigate the extent to which psychosomatic symptoms, i.e., psychological, musculoskeletal, and somatic complaints, predict high depressive and anxiety symptoms and diagnoses of depression and/or anxiety disorder 3 years and 6 years after the baseline.
2. To investigate the moderating roles of sex and SES in the associations between psychosomatic symptoms at baseline and depression and anxiety 3 and 6 years after baseline.

## Methods

### Study design

#### Procedure

This study was conducted using the longitudinal dataset from the “SALVe- Cohort” project [24]. This project aims at investigating the determinants of psychological and psycho-social development of two cohorts, born in 1997 and in 1999, following them from early adolescence to young adulthood. All individuals born in 1997 and 1999 and living in Västmaland Region in Sweden in 2012, were potentially eligible for the study. However, some individuals (e.g. those who had lived in Sweden for less than 5 years, and those with mental disabilities and severe illness) were originally excluded because of the inclusion criteria (see [24] for more details). Participants were contacted when they were 12-13 and 14-15 years-old (T1), 15-16 and 17-18 years-old (T2), and 18-19 and 20-21 years-old (T3). Västmanland County is viewed as fairly representative of Swedish society in terms of education, income, and employment levels, and also in terms of its distribution of urban and rural areas [25]. At T1, participants were contacted by regular mail and invited to participate in the longitudinal study. They were informed that their participation was voluntary and that they could interrupt it at any time. They returned a self-reported questionnaire at T1, T2 and T3. Participants were offered cinema tickets or gift cards (ca. 10 euro). which were funded with a grant from Svenska Spel Research Foundation, as incentives. The study was approved by Regional Ethical Board of Uppsala (Sweden) (Dnr. 2012/187).

### Participants

The original eligible adolescents were *N* = 4712, of whom *N* = 1868 (38.46%), responded at wave 1 (T1). At wave 2 (T2), the adolescents were contacted again, and 1575 (84%) of the original sample filled in the questionnaire (see [26]). Finally, 1174 (63%) of the adolescents who participated at T1 also returned the questionnaire at T3.

In sum, the final sample that participated at T1 and T2 consisted of 1575 young people, of whom 58% (*N* = 664) were female; 20% (*N* = 319) had non-Scandinavian parents; 49% (*N* = 804) were born in 1999 and 51% (*N* = 774) in 1997. The final sample that participated at T1 and T3 consisted of 1174 young people, of whom 61% (*N* = 722) were female; 20% (*N* = 229) had non-Scandinavian parents; 51% (*N* = 601) were born in 1999 and 49% (*N* = 573) in 1997.

### Measures

#### Psychosomatic symptoms

Psychosomatic symptoms were measured using eight items from the WHO scale assessing the frequency of symptoms in the last 3 months [22]. The answers range from never (0) to always (4). A Confirmatory Factor Analysis (CFA) showed that the model best fitting these items was from a second-order confirmatory factor analysis with a latent dimension of psychosomatic symptoms that comprised three subdimensions of symptoms, namely psychological (i.e., feeling nervous, feeling irritable, feeling sleepy, 3 items), somatic (headache, stomachache, 2 items) and musculoskeletal (pain in the shoulders/neck, pain in the back/hips, pain in the hands/knees/legs/feet, 3 items) (χ^2^ = 77.03, *p* > .01, df = 17, RMSEA = .04, CFI = .98, SRMR = .034). Accordingly, in the analysis we used both the total index of *Psychosomatic symptoms* (a = .71) and its three dimensions, namely *Psychological, Somatic, and Musculoskeletal symptoms.*

#### Depressive symptoms

The Depression Self-Rating Scale Adolescent version, DSRS-A [27] was used. The scale comprises 15 items based on the DSM-IV criteria for a major depressive disorder. The adolescents were asked about their depressive feelings in the last 2 weeks, with a yes/no response alternative. In accordance with the DSRS scale, the index used in the analysis was calculated by adding reported symptoms, where each set of symptoms was counted only once (0-9 points). Cronbach’s alphas were 0.81, 0.77 and 0.87, for T1, T2, and T3 respectively. DSM-IV A-criterion was used to create a dichotomous variable to distinguish between adolescents with and without a potential diagnosis of Major Depression Disorder (MDD). Adolescents that met the following two conditions: 1) the presence of at least one of the general criteria for depression (2 weeks of either dysphoric or irritable mood or loss of interest or pleasure in most activities); 2) the presence of at least four other symptoms, including sleep disturbances, weight loss or gain/appetite disturbances, psychomotor agitation or retardation, fatigue or loss of energy, feelings of worthlessness or guilt, concentration disturbances, and thoughts of suicide, were classified as with “high symptoms of depression”. We preferred to adopt this term rather than “diagnosis of MDD” because of the self-report nature of the measure. The cut off was validated in sensitivity and specificity studies [27, 28].

#### Anxiety

Anxiety symptoms were measured using the Spence Children’s Anxiety Scale (SCAS, [29]) at waves 1 and 2, while a short version of the same scale for adults was used at wave 3. The SCAS consists of 44 items, of which 38 cover all the six categories of anxiety disorder highlighted in the Diagnostic and Statistical Manual of Mental Disorders [30], while 6 are used as filler items to reduce negative bias. Alternative responses go from 0 (never) to 3 (always). The score of 33 has been identified as the cut-off for a diagnosis of generalized anxiety disorder [31] for children and adolescents. At wave 3, an adapted version with 15 items of the scale was used to assess the anxiety symptoms of the young adults. The 15 items were the following: 1) I worry that I will behave the wrong way and embarrass myself in front of others; 2) I suddenly feel as if I can’t breathe when there is no reason for this; 3) I worry about things a great deal; 4) I worry a lot more than most people; 5) I suddenly start to tremble or shake for no reason; 6) I worry about all sorts of different things; 7) I feel anxious in situations where I am the centre of attention, 8) I suddenly feel really scared when there is really nothing to be afraid of; 9) I find it difficult to stop worrying; 10) I feel afraid if I have to start a conversation with someone I don’t know; 11) I suddenly become dizzy or faint when there is no reason for this; 12) I feel tense or irritable; 13) My heart suddenly starts to beat too quickly for no reason; 14) I feel afraid that I will make a fool of myself in front of other people; 15) I feel nervous when I am introduced to new people. In the Swedish population, the cut-off for total anxiety disorder was > 18 (Spence, S., The Adult Anxiety Scale-15 (AAS-15). 2017, Personal communication). Cronbach alphas were 0.87, 0.89 and 0.91 and respectively. We used the above-mentioned cut-offs to classify the adolescents with low vs high symptoms.

#### Diagnoses of depressive and anxiety disorders

At T3, the young adults were asked whether they had received a diagnosis of depression and/or or some anxiety disorder by a physician, with yes/no as response alternatives.

#### Covariates

Sex (male, female), age (born in 1997 or 1999) and parents´ country of birth (Scandinavian parents vs at least one of the parents born outside Scandinavia) were used as covariates in the analyses.

### Statistical analyses

To investigate the effects of psychosomatic symptoms at T1 on levels of depression and anxiety at T2 and T3 and on diagnoses of depressive and/or anxiety disorder at T3, multivariate logistic models were constructed, controlling for initial levels of depression and anxiety respectively, and also for sex, age and SES. First, we ran models assessing the effect of the total index of psychosomatic symptoms on depressive or anxiety symptoms (low vs high, based on DSRS and SCASscutoffs) at T2 and T3, and on diagnoses of depression or anxiety disorder at T3. Then, we ran models using the subscales of psychosomatic symptoms, i.e., somatic, musculoskeletal, and psychological symptoms. Finally, to test whether sex was a moderator of the effects of psychosomatic symptoms, all the models were re-run adding an interaction term, i.e., psychosomatic symptoms * sex.

## Results

### Attrition analyses

Attrition analyses were conducted to investigate whether adolescents with mental health problems were more likely to drop out at T2 or at T3. We compared the means at T1 of adolescents who participated at T1 and T2 or at T1 and T3 with the means at T1 of adolescents who participated at T1 and not at T2, or at T1 and not at T3, respectively. We found no differences in psychosomatic (T1-T2: M = 9.53, sd = 4.92 vs M = 9.79, sd = 5.55 respectively, F (1, 1803) = .592, n.s., T1-T3: M = 9.57, sd = 4.97 vs M = 9.54, sd = 5.09 respectively, F (1,1803) = .013, n.s.), somatic (T1-T2: M = 2.61, sd = 1.55 vs M = 2.70, sd = 1.74 respectively, F (1, 1826) = .863, n.s., T1-T3: M = 2.63, sd = 1.57 vs M = 2.60, sd = 1.59 respectively F (1,1826) = .177, n.s.), musculoskeletal (T1-T2: M = 1.92, sd = 1.63 vs M = 1.97, sd = 1.71 respectively, F (1, 1800) = .275, n.s., T1-T3: M = 1.93, sd = 1.64 vs M = 1.92, sd = 1.64 respectively, F (1,1800) = .005, n.s.), or psychological symptoms (T1-T2: M = 2.72, sd = 1.50 vs M = 2.75, sd = 1.63 respectively, F (1, 1815) = .123, n.s., T1-T3: M = 2.72, sd = 1.48 vs M = 2.73, sd = 1.56 respectively, F (1,1815) = .027, n.s.). Moreover, adolescents who dropped out at T2 or at T3 were not more depressed (based on the DSRS cutoff) (T1-T2: χ^2^(df = 1, *N* = 1832) = 1.11, n.s.; T1-T3: χ^2^ (df = 1, *N* = 1819) = 3.32, n.s) or more anxious (based on the RCMA cutoffs) (T1-T2: χ ^2^(df = 1, *N* = 1832) = .09, n.s.; T1-T3: χ ^2^(df = 1, N = 1819) = .968, n.s.) at T1 than those that did not drop out.

### Descriptive analyses

The descriptive data are presented in Tables 1 and 2. At all the waves, girls reported higher psychosomatic symptoms in general, and also more specific somatic, psychological and musculoskeletal symptoms, than boys. They also reported higher percentages of depression and anxiety (see Tables 1 and 2). Finally, the percentage of internal missing data was very low ranging from 0 to 2%. Moreover, the listwise deletion method was used in the analyses, after checking with the Little’s MCAR test that data were missing completely at random [32].

**Table 1:** Means (standard deviations) and proportions (N) of the main variables of the study for the participants at T1 and T2

*Samples T1 and T2*	***Male*** ***% (N)//M (Sd)***	***Female*** ***%(N)//M (Sd)***	***F or χ2, df, and p***	***Total sample*** ***%(N)//M (Sd)***
***Independent variables***				
*Psychosomatic symptoms T1*	8.05 (4.50)	10.65 (4.95)	108.547 (1, 1552), *p* < .001	9.53 (4.92)
*Somatic symptoms T1*	2.10 (1.44)	2.97 (1.53)	132.47 (1, 1570), *p* < .001	2.60 (1.51)
*Psychological symptoms T1*	2.33 (1.38)	3.00 (1.51)	79.27 (1, 1563), *p* < .001	2.71 (1.49)
*Musculoskeletal symptoms T1*	1.66 (1.55)	2.10 (1.66)	28.24 (1, 1550), *p* < .001	1.91 (1.63)
*High anxiety symptoms (RCMAS) T1*	5% (35)	19% (169)	60.31, df = 1, *p* < .001	13% (204)
*High depressive symptoms (DSRS) T1*	6% (42)	16%(148)	318.84; df = 1, *p* < .001	12% (190)
***Dependent variables***				
*High anxiety symptoms (RCMAS) T2*	6.5% (43)	33% (293)	152.22, df = 1, *p* < .001	21% (336)
*High depressive symptoms (DSRS) T2*	14% (91)	33% (296)	74.64 (1), *p* < .001	25% (387)

**Table 2:** Means (standard deviations) and proportions (N) of the main variables of the study for the participants at T1 and T3

Samples T1 and T3	Male %(N)//M (Sd)	Female %/(N)//M (Sd)	F or χ2, df, and p	Total sample %/(N)//M (Sd)
**Independent variables**				
*Psychosomatic symptoms T1*	8.07 (4.62)	10.52 (4.97)	69.84 (1, 1151), *p* < .001	9.57 (4.97)
*Somatic symptoms T1*	2.12 (1,44)	2.95 (1.53)	81.91 (1, 1169), *p* < .001	2.63 (1.57)
*Psychological symptoms T1*	2.98 (1.49)	3.00 (1.51)	59.15 (1, 1157), *p* < .001	2.71 (1.49)
*Musculoskeletal symptoms T1*	1.66 (1.58)	2.09 (1.66)	18.51 (1, 1148), *p* < .001	1.93 (1.64)
*High anxiety symptoms (RCMAS) T1*	5% (24)	17% (122)	33.99, df = 1, *p* < .001	13% (147)
*High depressive symptoms (DSRS) T1*	6% (27)	15% (106)	20.95; df = 1, *p* < .001	11% (133)
***Dependent variables***				
*High anxiety symptoms (RCMAS) T3*	16% (71)	37% (264)	59.52, df = 1, *p* < .001	29% (336)
*High depressive symptoms (DSRS) T3*	25% (112)	44% (317)	44.09 (1), *p* < .001	37% (430)
*Diagnosis of depression T3*	3% (16)	9% (68)	14.57 (1), *p* < .001	7% (83)
*Diagnosis of anxiety T3*	3% (15)	9% (68)	15.86, df = 1, *p* < .001	7% (83)

### Psychosomatic symptoms at T1 and depression and anxiety at T2 and T3

Higher number of psychosomatic symptoms at T1 increased the risk for high depressive symptoms at T2 (OR = 1.06, 95% CI: 1.02-1.09) and T3 (OR = 1.06, 95% CI: 1.03-1.09), after controlling for the initial values of depressive symptoms, sex, parents´ country of birth and age. When the symptoms were analyzed separately, it emerged that musculoskeletal symptoms drove this relation. That is, youth with a high number of musculoskeletal symptoms showed higher risk for high depressive symptoms at T2 (OR = 1.10, 95% CI: 1.01-1.19), and T3 (OR = 1.13, 95% CI: 1.14-1.24), after controlling for initial values of depressive symptoms, sex, parents´ country of birth and age. Somatic and psychological symptoms were not significant (see Table 3).

**Table 3:** Odds ratios (OR) for the associations between mental health problems at baseline and depression at T2 and T3, controlling for sex, age and SES

	High depressive symptoms T2	High depressive symptoms T3	Diagnosis of depressive disorder T3
***Model 1***	**OR (95% CI)**^**a**^	**OR (95% CI)**^**b**^	**OR (95% CI)**^**c**^
*Psychosomatic symptoms*	1.06 (1.02-1.09)	1.06 (1.03-1.09)	1.11 (1.04-1.17)
*Number of depressive symptoms at T1*	1.30 (1.22-1.39)	1.13 (1.05-1.21)	1.18 (1.06-1.33)
*Sex*	2.29 (1.73-3.04)	1.90 (1.45-2.49)	2.02 (1.13-3.60)
*Age*	.95 (.83-1.08)	1.11 (.97-1.25)	1.17 (.92-1.49)
*Parents´ country of birth*	1.14 (.83-1.56)	.93 (.67-1.30)	.47 (.23-.99)
***Model 2***	**OR (95% CI)**^**d**^	**OR (95% CI)**^**e**^	**OR (95% CI)**^**f**^
*Somatic symptoms*	1.09 (.99-1.21)	1.06 (.96-1.17)	1.40 (1.17-1.68)
*Musculoskeletal symptoms*	1.10 (1.01-1.19)	1.13 (1.04-1.24)	1.03 (.88-1.19)
*Psychological symptoms*	1.04 (.93-1.16)	1.05 (.94-1.18)	1.11 (.91-1.36)
*Number of depressive symptoms*	1.31 (1.22-1.40)	1.13 (1.06-1.21)	1.18 (1.05-1.32)
*Sex*	2.29 (1.72-3.05)	1.89 (1.43-2.49)	1.88 (1.04-3.36)
*Age*	.95 (.83-1.06)	1.12 (.98-1.26)	1.18 (.93-1.51)
*Parents´ country of birth*	1.12 (.81-1.55)	.95 (.68-1.32)	.51 (.24-1.07)

Δ Nagelkerke R^2^ between the models without and with psychosomatic symptoms

^a^ Δ Nagelkerke *R*^*2*^ = .03

^b^ Δ Nagelkerke *R*^*2*^ = .03

^c^ Δ Nagelkerke *R*^*2*^ = .03

^d^ Δ Nagelkerke *R*^*2*^ = .03

^e^ Δ Nagelkerke *R*^*2*^ = .03

^f^ Δ Nagelkerke *R*^*2*^ = .03

Regarding the diagnosis of depressive disorders, adolescents who reported high levels of somatic symptoms at T1 showed an increased probability (OR = 1.40, 95% CI: 1.17-1.68) of a diagnosis of depression at T3, even after controlling for depressive symptoms at baseline.

Psychosomatic symptoms at T1 did not increase the odds of showing high anxiety symptoms at T2, but they did at T3 (OR = 1.05, CI = 1.02-1.08) (see Table 4). Youth with high levels of somatic symptoms at T1 had increased odds (OR = 1.18, 95% CI: 1.06-1.32) of suffering from a high level of anxiety symptoms at T3 (see Table 4).

**Table 4:** Odds Ratios (OR) for the associations between mental health problems at baseline and anxiety at T2 and T3, controlling for sex, age and SES

	High anxiety symptoms T2	High anxiety symptoms T3	Diagnosis of anxiety symptoms T3
***Model 1***	**OR (95% CI)**^**a**^	**OR (95% CI)**^**b**^	**OR (95% CI)**^**c**^
*Psychosomatic symptoms*	1.02 (.98-1.05)	1.05 (1.02-1.08)	1.13 (1.07-1.19)
*Number of anxiety symptoms at T1*	1.08 (1.06-1.09)	1.04 (1.03-1.06)	1.01 (1.00-1.03)
*Sex*	4.31 (3.00-6.27)	2.05 (1.49-2.81)	2.21 (1.19-4.08)
*Age*	1.13 (.98-1.31)	1.12 (.98-1.29)	1.11 (.87-1.40)
*Parents´ country of birth*	1.16 (.81-1.66)	.76 (.52-1.11)	.50 (.24-1.05)
***Model 2***	**OR (95% CI)**^**d**^	**OR (95% CI)**^**e**^	**OR (95% CI)**^**f**^
*Somatic symptoms*	1.02 (.91-1.14)	1.18 (1.06-1.32)	1.30 (1.08-1.57)
*Musculoskeletal symptoms*	1.02 (.93-1.13)	1.01 (.92-1.12)	1.12 (.96-1.31)
*Psychological symptoms*	1.04 (.92-1.17)	1.05 (.93-1.18)	1.12 (.91-1.38)
*Number of anxiety symptoms*	1.08 (1.06-1.09)	1.04 (1.03-1.06)	1.02 (1.00-1.04)
*Sex*	4.39 (3.01-6.43)	1.94 (1.42-2.68)	2.22 (1.18-4.17)
*Age*	1.12 (97-1.30)	1.12 (.98-1.30)	1.10 (.86-1.40)
*Parents’ country of birth*	1.13 (.78-1.63)	.79 (.52-1.19)	.46 (.21-1.00)

Δ Nagelkerke R^2^ between the models without and with psychosomatic symptoms

^a^ Δ Nagelkerke *R*^*2*^ = .06

^b^ Δ Nagelkerke *R*^*2*^ = .03

^c^ Δ Nagelkerke *R*^*2*^ = .03

^d^ Δ Nagelkerke *R*^*2*^ = .06

^e^ Δ Nagelkerke *R*^*2*^ = .03

^f^ Δ Nagelkerke *R*^*2*^ = .03

Regarding the diagnosis of anxiety disorder, psychosomatic symptoms at T1 increase the probability (OR = 1.13, CI = 1.07-1.19) of an anxiety disorder at T3. Moreover, reporting somatic symptoms at T1 was associated with an increased probability (OR = 1.30, 95% CI: 1.08-1.57) of a diagnosed anxiety disorder at T3.

### The moderating effect of sex

Sex did not moderate the relation between psychosomatic symptoms at T1 and depression at T2 or at T3. When it comes to the different subscales of symptoms, none of them had a different effect on depression in boys and girls (see Table 5).

**Table 5:** Odd ratios (OR) and Cohen D (d) of the interaction terms used to assess the moderating role of sex in predicting the impact of mental health problems at baseline on depression and anxiety at T2 and T3

***Models with depression as an outcome***	***High depressive symptoms T2***	***High depressive symptoms T3***	***Diagnosis of depression disorder T3***
	**OR (95% CI)**	**OR (95% CI)**	**OR (95% CI)**
*Psychosomatic symptoms x sex*	.98 (.93-1.04)	1.01 (.96-1.07)	1.03 (.92-1.15)
*Somatic symptoms x sex*	1.08 (.90-1.29)	1.13 (.95-1.34)	1.12 (.79-1.58)
*Musculoskeletal symptoms x sex*	.95 (.81-1.12)	.97 (.82-1.14)	1.15 (.83-1.59)
*Psychological symptoms x sex*	.90 (.74-1.09)	1.09 (.90-1.33)	.91 (.61-1.35)
***Models with anxiety as outcome***	***High anxiety symptoms T2***	***High anxiety symptoms T3***	***Diagnosis of anxiety disorder T3***
	**OR (95% CI)**	**OR (95% CI)**	**OR (95% CI)**
*Psychosomatic symptoms x sex*	.97 (.90-1.05)	.98 (.92-1.05)	.98 (.86-1.11)
*Somatic symptoms x sex*	1.06 (.83-1.36)	1.08 (.87-1.35)	.86 (57-1.28)
*Musculoskeletal symptoms x sex*	.98 (.78-1.22)	.93 (.77-1.13)	1.03 (.71-1.50)
*Psychological symptoms x sex*	.81 (.62-1.04)	1.02 (.73-1.17)	.94 (.62-1.44)

Sex did not moderate the relation between psychosomatic symptoms at T1 and anxiety at T2 or T3. Moreover, none of the different subscales of symptoms had a different relation to anxiety in boys and girls (see Table 5).

## Discussion

The aim of the current study was to investigate whether an adolescent’s mental health problems, in term of psychosomatic symptoms, among Swedish youth, can predict an increased risk of developing depressive and anxiety symptoms and/or receiving the diagnosis of a depressive or anxiety disorder in late adolescence and early adulthood. We found that psychosomatic symptoms in adolescence increased the risk of both depression and anxiety three and 6 years later. Moreover, when looking into the types of symptoms that were most predictive, we found that Swedish adolescents who suffer from musculoskeletal symptoms are more at risk of developing high depressive symptoms, while Swedish adolescents who complain of somatic symptoms, e.g., headaches and stomachaches, have an increased risk of being diagnosed with an anxiety and/or depressive disorder 6 years later. Finally, these effects did not differ between girls and boys.

Our study shows that somatic symptoms, such as headaches and stomachaches, can predict depression and/or an anxiety disorder up to 6 years later. This result is in line with many cross-sectional studies that have pointed to relations between such symptoms and depression and anxiety [13, 33], even to the extent that it has been argued that these symptoms can be used as predictors of suicide attempts [34]. There are also some longitudinal studies that have come to the same conclusion, showing that somatic symptoms independently predict both depression and anxiety, after controlling for depression and anxiety at baseline and using both diagnostic interviews and registry data on hospital-based mental health care [14, 20, 23]. The present study corroborates this assertion, while adding that the contributions of both psychosomatic symptoms in general and of somatic symptoms in particular to depression and anxiety are the same for girls and boys. To the best of our knowledge, this is the first study to determine that, despite the different levels of mental health problems in girls and boys, somatic symptoms play a similar role in the development of depressive and anxiety disorders in adulthood.

Another important contribution of the study lies in its focus on the role of psychosomatic symptoms other than the somatic, i.e., on musculoskeletal symptoms. Although some studies have shown that musculoskeletal pain in children is associated with internalizing symptoms [35], to our knowledge, the long-term effects of symptoms of this kind have not been investigated before. We found that they may also contribute to a deterioration of mental health, with an increase in depression in late adolescence and young adulthood. When assessed via self-report measures, musculoskeletal symptoms were the only symptoms in the current study that were able to predict depression three and 6 years later. This result highlights the importance of not underestimating the role of these symptoms. While somatic symptoms have been investigated extensively, and many hypotheses have been advanced regarding their relations with psychiatric disorders (for a review, see 16), the reasons why musculoskeletal symptoms might be associated with mental health problems are unknown. The symptoms may be a direct consequence of increased computer time in new generations of youth [36] entailing decreased physical activity, which in turn is associated with reduced mental health, whether or not in the form of diagnosed problems [37]. This might provide an explanation for why this association has not involved real-life diagnoses of depressive and anxiety disorders of the kind our study has highlighted. However, the hypothesis needs to be confirmed in future studies.

### Limitations and strengths

The study has some limitations. First, the psychosomatic symptoms were assessed only at baseline and not at the follow-ups. This limits the opportunity to understand how they might develop in late adolescence and young adulthood, and their contribution to possible mental ill-health. Moreover, as in all the other study population studies in this arena, initial acceptance of participation in the study was somewhat low (around 40%). Although this rate is quite common in this type of study, we cannot exclude the possibility that low participation impacted the external validity of the results, undermining the representativeness of the sample, especially when considering that non-participants are often more at risk of the negative outcomes considered (e.g., depression). Attrition, however, is unlikely to have influenced the results as the adolescents who stayed in the project did not differ from those who dropped out at T1. Finally, using the psychosomatic symptoms as a continuous variable makes it more difficult to interpret the transformation of OR to Cohen’s D because the effect size depends on the scale. Thus, we were unable to conduct this transformation. Further, the lack of validated cut-offs in the psychosomatic symptoms variable prevented us to use it as dichotomous. Finally, listwise deletion can be problematic in a scenario with many variables even with low missing values and MCAR data. However, in our models the percentage of missing values never exceeds 3.1% in total, thus we assess the likelihood for bias as low.

This study also has some strengths. First, to the best of our knowledge, it is the first study to examine the longitudinal effects of different subcategories of psychosomatic symptoms on mental health. Second, the use of different measures to assess mental health problems, i.e., self-reported symptoms and clinical diagnoses, provide good support for the validity of the results.

## Conclusions

Many recent studies have pointed to an increase in mental health problems, often in terms of psychosomatic symptoms, in the new generations of youth. This study demonstrates that high levels of psychosomatic symptoms in adolescence increase the risk of developing high levels of both depressive and anxiety symptoms and depressive and anxiety disorders. Therefore, it provides a rationale closely to monitor adolescents, boys and girls to the same extent, and especially those with somatic and musculoskeletal symptoms, in order to prevent the development of serious disorders in late adolescence and early adulthood.

## Data Availability

The datasets used and/or analysed during the current study are available from the corresponding author on reasonable request.

## Abbreviations

DSRS: The Depression Self-Rating Scale Adolescent version
SCAS: Spence Children’s Anxiety Scale
SES: Socio-economic status
